# Synthesis, spectroscopic and crystallographic analysis of the Zn-complex of a di(*β*,*β′*-sulfoleno)pyrrin: model for Zn-complexes of bilirubin and of phylloxanthobilins

**DOI:** 10.1007/s00706-016-1748-0

**Published:** 2016-04-21

**Authors:** Chengjie Li, Klaus Wurst, Yaqing Feng, Bernhard Kräutler

**Affiliations:** Institute of Organic Chemistry and Centre of Molecular Biosciences, University of Innsbruck, 6020 Innsbruck, Austria; School of Chemical Engineering and Technology, Tianjin University, Tianjin, 300072 People’s Republic of China; Institute of General, Inorganic and Theoretical Chemistry, University of Innsbruck, 6020 Innsbruck, Austria

**Keywords:** Crystal structure, Dipyrrin, Fluorescence spectroscopy, Coordination chemistry, Zn-complex

## Abstract

**Abstract:**

A high yield preparation, spectroscopic and crystallographic investigation of the crystalline Zn-complex of a di(*β*,*β′*-sulfoleno)pyrrin are reported here. In the brightly green fluorescent Zn-complex of the hardly luminescent di(*β*,*β′*-sulfoleno)pyrrin, the metal ion is bound by two di(*β*,*β′*-sulfoleno)pyrrin ligands, as revealed first by its mass spectra. The crystal structure of this Zn-complex of the di(*β*,*β′*-sulfoleno)pyrrin confirmed a regular 2:1 composition of the bidentate di(*β*,*β′*-sulfoleno)pyrrin ligand and the metal ion. The latter was coordinated in a distorted tetrahedral fashion, as found in other dipyrrin Zn-complexes. The here studied Zn-complex of a designed di(*β*,*β′*-sulfoleno)pyrrin ligand provides insights into the coordination properties of the proposed (2:1)- and (2:2)-complexes of phylloxanthobilin and bilirubin, respectively, which are two abundant natural bilin-type tetrapyrroles.

**Graphical abstract:**

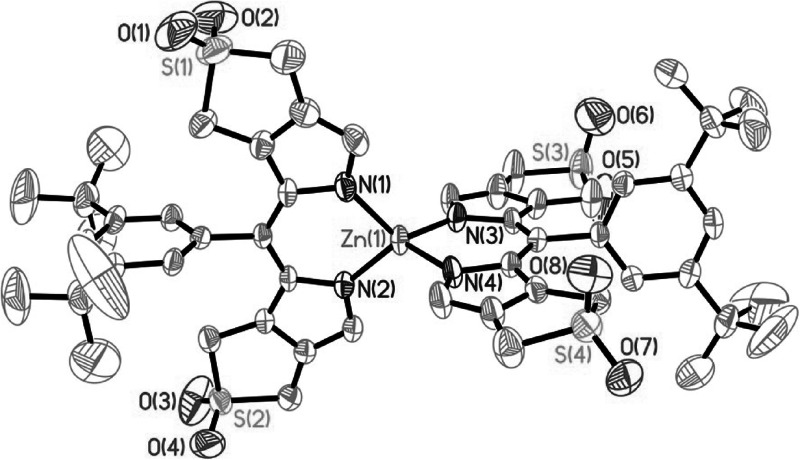

## Introduction

Dipyrrins (or dipyrromethenes) feature two conjugated pyrrole rings and represent (formal) dipyrrolic building blocks for the construction of porphyrins and related tetrapyrrolic macrocycles [1]. The complexes of the bidentate dipyrrins with boron (the ‘BODIPY’-complexes) [2] or with transition metal ions [3, 4] have attracted particular attention due to the ‘predictable’ coordination properties of dipyrrins, and the tunable emission and absorption properties of their metal complexes [5–9]. Hence, the design of dipyrrin ligands has been attractive, and dipyrrin chemistry has taken advantage of the development of a broad range of strategies for their construction [2–5, 7, 8]. In one approach (used here), dipyrrins are prepared by dehydrogenation of easily accessible corresponding dipyrromethanes [3, 4].

In the context of our recent interest in the metal coordination properties of yellow chlorophyll catabolites (phylloxanthobilins) [10], and of other natural linear tetrapyrroles derived from chlorophyll [11, 12], we report here on our investigations of a model dipyrrole, the di(*β*,*β′*-sulfoleno)pyrrin **2**.

## Results and discussion

**Fig. 1:**
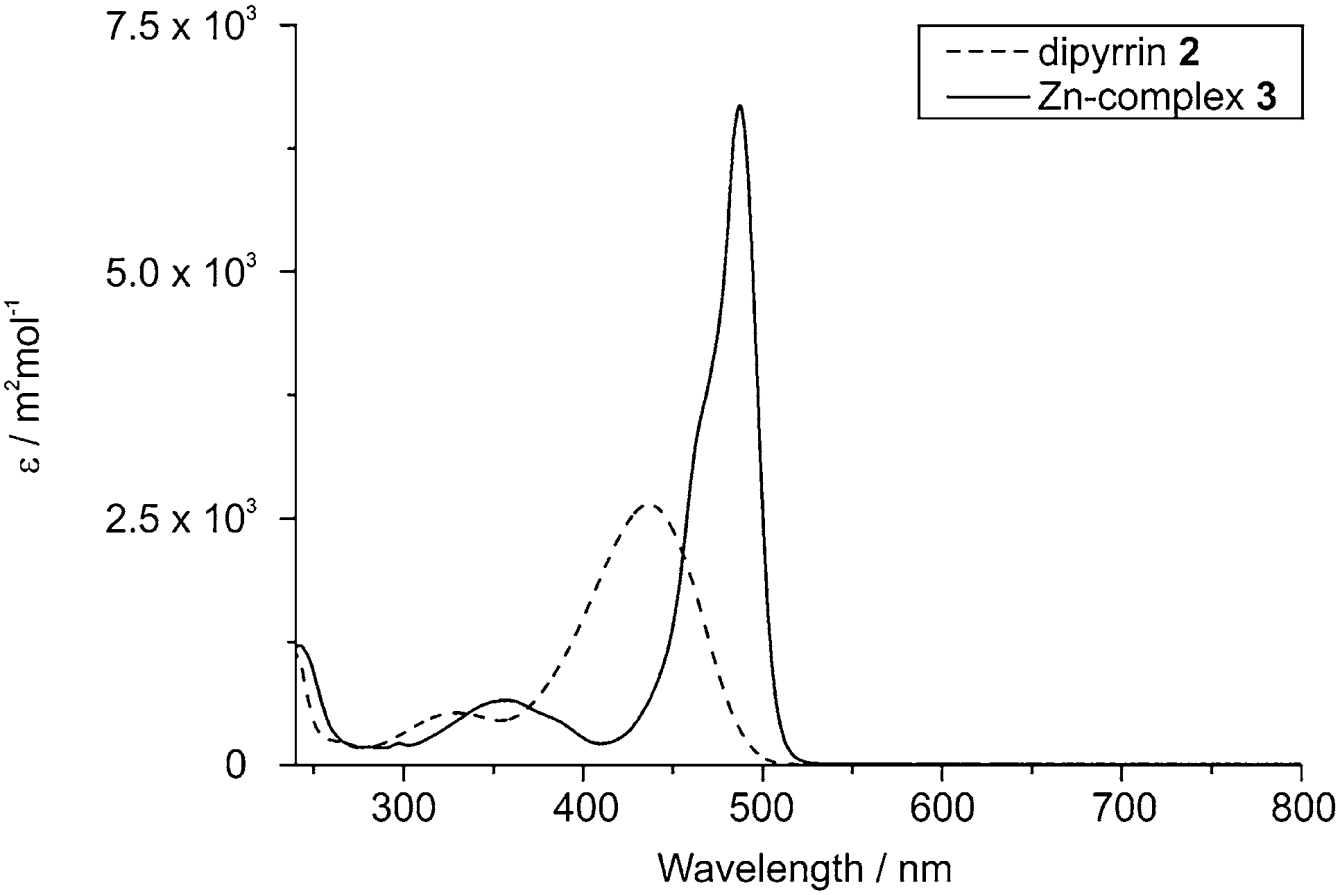
UV–Vis spectra of the dipyrrin **2** and of its Zn-complex **3** (=Zn-(**2**)_2_) in CH_2_Cl_2_ (4 × 10^−6^ mol/dm^3^)

**Fig. 2:**
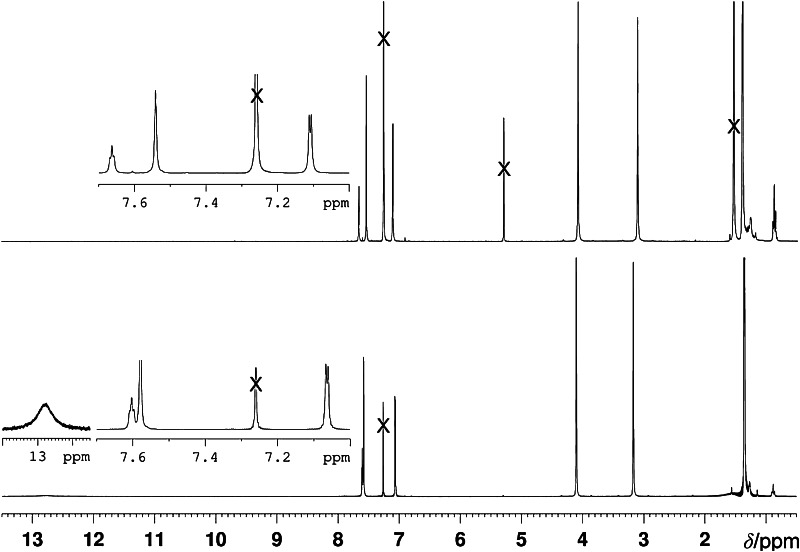
^1^H NMR spectra of the dipyrrin **2** (*bottom*) and of its Zn(II)-complex **3** (Zn-(**2**)_2_) (*top*) in CDCl_3_ (300 MHz, 25 °C, × = solvent signals)

Our synthetic route to di(*β*,*β′*-sulfoleno)pyrrin **2** relied on the earlier made corresponding di(*β*,*β′*-sulfoleno)pyrromethane (**1**), available, in turn, from condensation of 3,5-di-*tert*-butylbenzaldehyde and *β*,*β′*-sulfolenopyrrole [13]. Dipyrromethane **1** was oxidized with dicyanodichlorobenzoquinone (DDQ) to furnish bright yellow **2** in 76 % yield, after crystallization in CH_2_Cl_2_/*n*-C_6_H_14_ (see Scheme 1). The UV/Vis spectrum of the dipyrrin **2**, displayed in Fig. 1, exhibits characteristic maxima at 436.5 and 327 nm, comparable to the one of bilirubin [14, 15], or of a recently described yellow chlorophyll catabolite (YCC, a phylloxanthobilin) [16, 17]. A FAB-mass spectrum featured a strong pseudo-molecular ion at *m/z* = 513.1 [M + H]^+^, confirming its molecular formula as C_27_H_32_N_2_O_4_S_2_. Fragments at *m/z* = 448.2 and 384.2 indicated consecutive loss of the two SO_2_-groups. The ^1^H NMR spectrum of **2** exhibited two singlets at intermediate field of the two pairs of symmetry equivalent *β*-methylene groups, a singlet at 7.58 ppm of the pyrrole-α positions, the signals of aryl *o*- and *p*-protons at low field, and a broad signal of an NH at 12.76 ppm (see Fig. 2, bottom).

**Figure:**
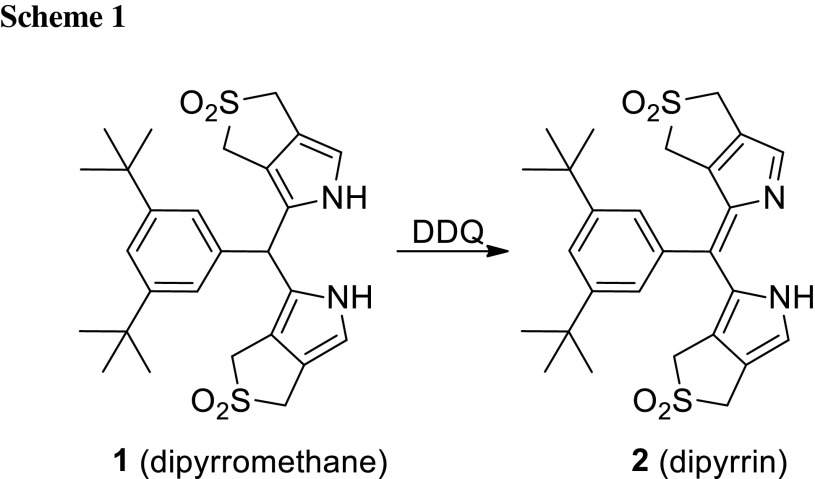

For the preparation of **3**, a solution of 15 mg of Zn(OAc)_2_ 2H_2_O (68.4 μmol, 18 eq) in 0.3 cm^3^ MeOH was mixed into a solution of 2 mg **2** (3.8 μmol) in 2.7 cm^3^ of CH_2_Cl_2_ at room temperature. After 5 min, the reaction mixture was worked up by extraction and evaporation of the solvent (see “Experimental” part). The Zn-complex **3** crystallized from CH_2_Cl_2_/*n*-C_6_H_14_ as pink-red crystals (2.0 mg, 94 % yield).

The molecular formula of the dipyrrin Zn-complex **3** was indicated as C_54_H_62_N_4_O_8_S_4_Zn from analysis of its pseudo-molecular ion [M+H]^+^ at *m/z* = 1087.2. Corresponding significant fragments occurred at *m/z* = 1023.3, 894.4, and 830.4, due to consecutive loss of the one, three, and four SO_2_-groups, respectively. The derived molecular formula of the Zn-complex **3** suggested the presence of two dipyrrin ligands **2** and one Zn(II)-ion, i.e. to represent Zn-(**2**)_2_ (see Scheme 2).

**Figure:**
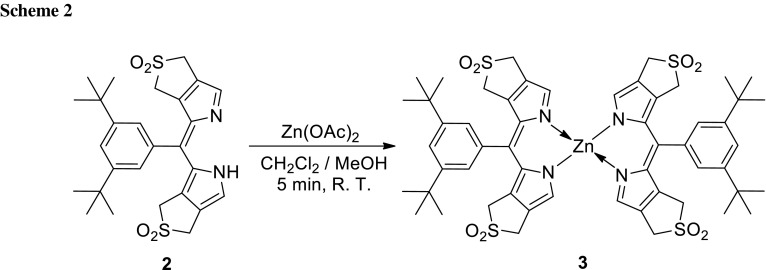

**Fig. 3:**
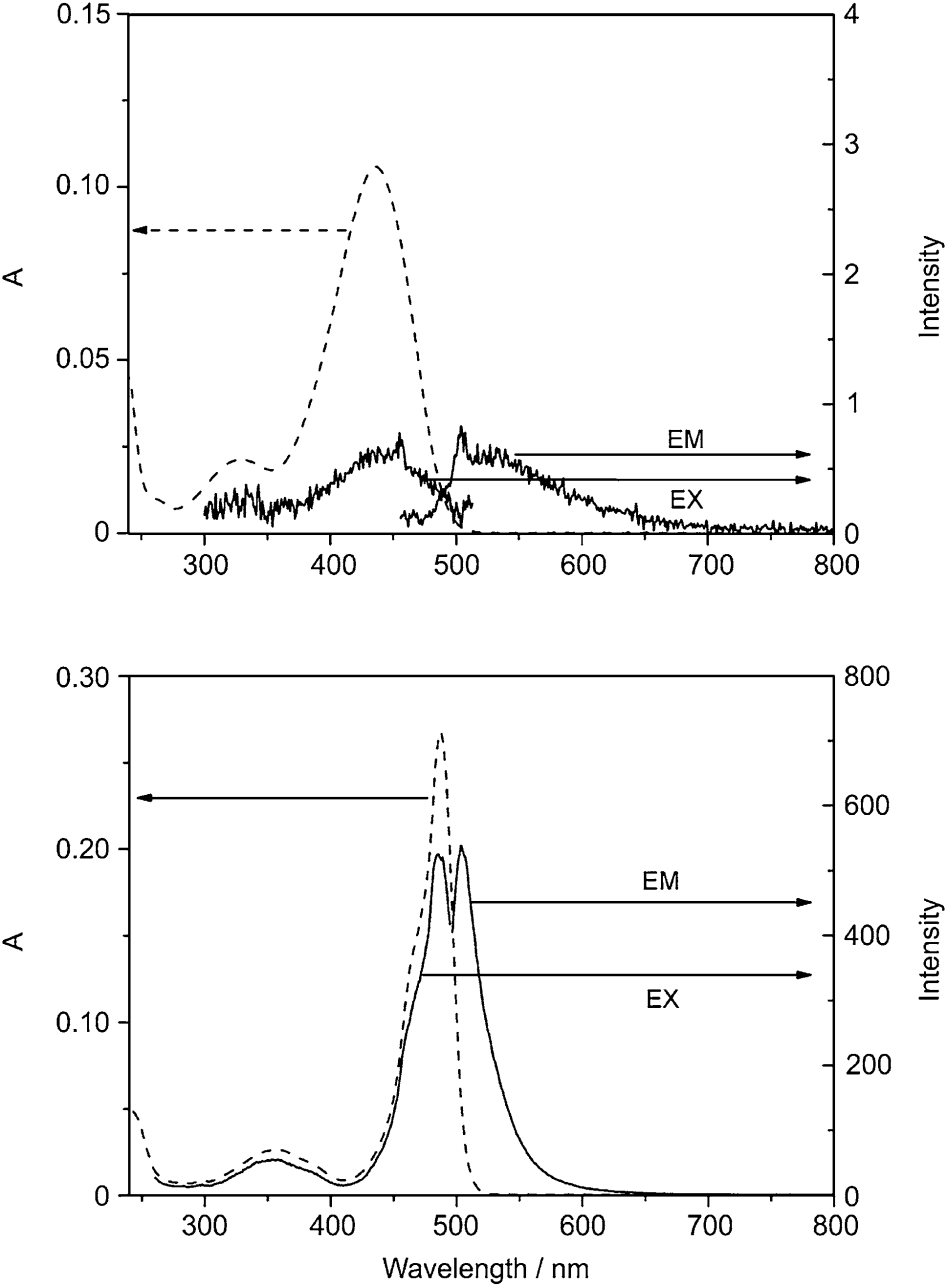
Fluorescence spectra of **2** and **3** in CH_2_Cl_2_ (*top*: **2**, 4 × 10^−6^ mol/dm^3^, EM: excited at 436 nm, EX: observed for 530 nm; *bottom*: **3**, 4 × 10^−6^ mol/dm^3^, EM: excited at 487 nm, EX: observed for 505 nm)

The pink-red Zn-complex **3** displayed a UV/Vis-spectrum in CH_2_Cl_2_ that featured a maximum at 487 nm (and a shoulder at 463 nm), corresponding to a 51 nm bathochromic shift, when compared with the spectrum of the dipyrrin **2** (see above). Similar bathochromic shifts of the absorption spectrum upon coordination of a Zn(II) ion were observed in Zn-complexes of bilirubin [18], or of phylloxanthobilins [10, 12]. Analysis of the Zn-complex **3** by fluorescence spectroscopy showed an intense emission band at 505 nm, whereas the metal-free dipyrrin **2** displayed little luminescence with a maximum around 530 nm (see Fig. 3). The excitation spectrum of dipyrrin Zn-complex **3**, observed at 505 nm, fitted the absorption spectrum of **3**. As, in contrast, the dipyrrin **2** was essentially non-luminescent in CH_2_Cl_2_, the rapid coordination of Zn ions and formation of **3** lightened up an intense green luminescence with about 200–300-fold higher intensity (see Fig. 3).

In the ^1^H NMR spectrum (in CDCl_3_, see Fig. 2) of the Zn-complex **3** [Zn-(**2**)_2_], a signal of an HN was lacking (which was found at 12.76 ppm in the spectrum of the dipyrrin **2**), consistent with bidentate binding of the dipyrrin ligand **2** to the coordinated Zn(II) ion. Signals of aryl-*o* hydrogens and aryl-*p* hydrogen are slightly shifted to lower field while signals of *β*-methylene groups and pyrrole-*α* hydrogens are slightly moved to higher field.

**Fig. 4:**
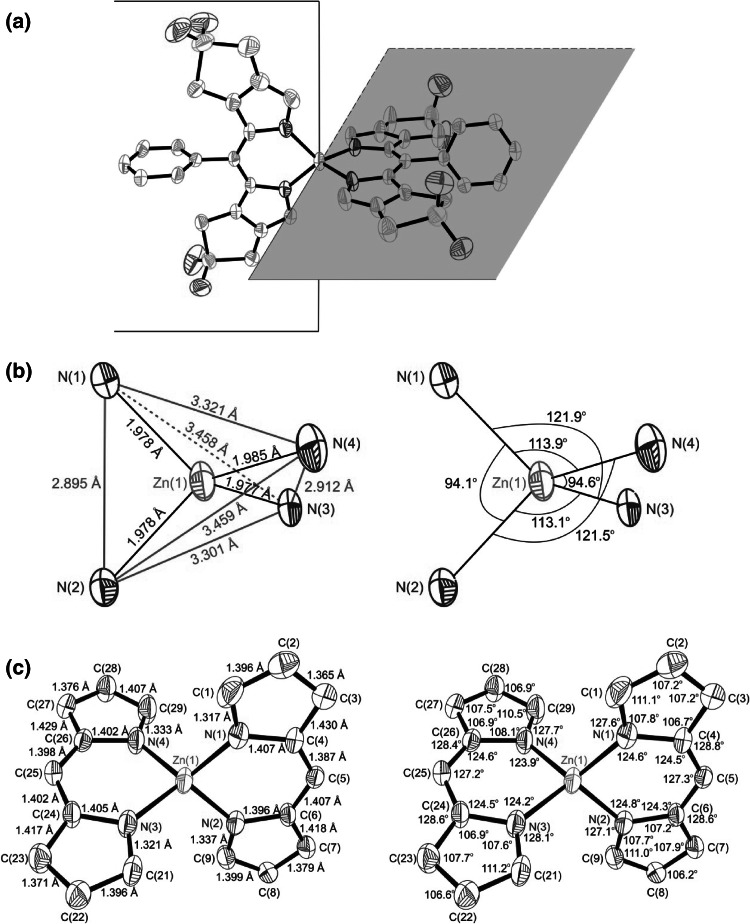
ORTEP-plots of the crystal structure of the bis(dipyrrinato) Zn-complex **3**. **a** Model of the structure of **3** highlighting the two planes spanned by the dipyrrin ligands (H atoms and tert-butyl substituents at meso-aryl groups are omitted). **b** Model of the distorted tetrahedral core of **3**, with specified lengths Zn-N bonds and distances between the N’s (*left* part) and N–Zn–N bond angles of the Zn–N_4_ core (*right* part). **c** Bond lengths (*left*) and bond angles (*right*) in the dipyrrin cores of **3**

X-ray diffraction quality crystals of the Zn-complex **3** grew from a solution of **3** in CH_2_Cl_2_ when *n*-C_6_H_14_ was mixed in slowly at 4 °C. The Zn-complex **3** crystalized in the triclinic system with space group *P*-1 (no. 2). A unit-cell contained four molecules of **3**. The crystal structure of **3** showed two bidentate dipyrrin moieties wrapped around one Zn(II) center leading to coordination in a distorted tetrahedral fashion with N–Zn–N angles of about 94, 113, and 121° (Fig. 4). The bonds of the four N atoms to the coordinated Zn ion are 1.98 Å long, consistent with crystallographic data from other Zn-dipyrrin complexes (1.96–1.98 Å) [19–21]. The structure of **3** in the triclinic crystal deviates slightly from the symmetric model reported for crystals of other bis(dipyrrinato) Zn-complexes [19]. The planes of the two dipyrrin ligands are roughly vertical to each other (82° dihedral angle), as are the aryl groups at the 5-position with respect to the conjugated pyrrole system in the same ligand moiety (85°). Thus, the plane defined by one aryl group at the meso-position is observed at 3.8° with respect to the plane of the conjugated pyrrole system in the other ligand. In contrast, the other aryl group at 5-position is inclined by 17.1° with respect to the conjugated pyrrole system in the second dipyrrin unit. Probably, these small symmetry-deviations are consequences of the packing in the triclinic crystal. The bond lengths and bond angles within the dipyrrin ligands in **3** are similar to those of other bis(dipyrrinato) Zn-complexes [19–21]. The crystal structure of **3** reflects the symmetric, bidentate coordination behavior of the dipyrrin **2**. Thus, the Zn-complex **3** may represent a valuable model for the chelation pattern in non-crystalline Zn(II)-complexes of natural oligopyrroles with similar chromophores, e.g. of bilirubin [18] or of the phylloxanthobilin YCC [10].

## Conclusion

Dipyrrin **2** is a yellow dipyrrole that possesses two conjugated pyrrolic rings and shows negligible luminescence. It may be considered a simple model compound for the chromophore part of some natural tetrapyrroles, such as the heme-derived bilirubin (BR) [14, 15] and phylloxanthobilins [17] or yellow chlorophyll catabolites (YCCs) [10, 16]. Binding of Zn(II) ions to the bidentate **2** furnishes the 2:1 complex **3**, the crystal structure of which exhibited a distorted tetrahedral structure. This type of coordination pattern was derived for the (2:1)-complex of a YCC with Zn(II) ions [10]. Similar, furthermore, to observation with the Zn(II)-complex of the YCC [10], the bis(dipyrrinato) Zn-complex **3** displays intensive green luminescence. Hence, the present study helps to model the coordination behavior of Zn-complexes of natural oligopyrroles with similar conjugated chromophores, e.g. of BR [18] or of YCC [10, 12], and to gain basic insights into their luminescence properties. Bis(dipyrrinato) Zn-complexes, and related boron complexes of dipyrrins (BODIPYs) [2], exhibit intensive and tunable absorption and emission properties, which make them useful in various optical applications [5–7, 9]. In contrast to BODIPYs, bis(dipyrrinato) Zn-complexes exhibit a tetrahedral coordination pattern in 2:1 assemblies (ligand: Zn), giving them considerable potential in supramolecular structuring [21, 22]. The sulfoleno-units of the dipyrrin **2** and of the bis(dipyrrinato) Zn-complex **3** are, furthermore, ‘programmed’ for introduction of covalent modifications at the pyrrole *β*-positions by [4+2]-cycloaddition reactions. As was recently developed with porphyrinoids, such as tetra-sulfolenoporphyrins [23–25] and a tetra-sulfolenocorrole [13], the dipyrrinato-units of **2** and **3** could, thus, also open up efficient access to further designed functionalized supramolecular assemblies.

## Experimental

Dichlorodicyano-*p*-benzoquinone (DDQ) and zinc acetate dihydrate [Zn(OAc)_2_ 2H_2_O] were reagent grade commercial chemicals from Fluka and were used as received; EtOAc, dichloromethane, methanol (MeOH), and *n*-C_6_H_14_ were from Acros and were distilled before use. Column chromatography (CC): Fluka silica gel 60 (230–400 mesh). Thin layer chromatography (TLC): Merck 0.25 mm silica gel 60 plates. Equipment: UV/Vis: Agilent Cary 60 UV–Visible, *λ*_max_ in nm (log *ε*). Fluorescence (FL): Varian Cary Eclipse, *λ* in nm (rel. intensity); Nuclear magnetic resonance (^1^H) spectra: Bruker 300 at 298K, chemical shifts (*δ*) in ppm, with *δ* (CHCl_3_) = 7.26 ppm, signal assignment follows the X-ray numbering scheme. FAB-MS: Finnigan MAT-95, positive ion mode, NOBA matrix; X-ray analyses: data collection on a Nonius Kappa CCD, equipped with graphite mono-chromatized Mo-Kα-radiation (*λ* = 0.71073 Å) at 233K. Melting point: Büchi 535.

### *5*-*(3,5*-*Di*-*tert*-*butylphenyl)*-*di(β,β′*-*sulfoleno)pyrrin* (**2**, C_27_H_32_N_2_O_4_S_2_)

To the solution of 2 mg dipyrromethane **1** (3.8 μmol) [13] in 1 cm^3^ CH_2_Cl_2_ 1.4 mg DDQ (6.2 μmol, 1.6 equiv) was added. After 20 h at room temperature, the reaction mixture was diluted to 20 cm^3^ with CH_2_Cl_2_ and washed with saturated aq. NaHCO_3_ (3 × 15 cm^3^). The organic phase was filtered through a plug of dry cotton-wool and evaporated to dryness under reduced pressure to give a brown residue. The residue was dissolved in 1.5 cm^3^ CH_2_Cl_2_ and loaded on a silica gel column (1.5 × 10 cm). The product was washed down with CH_2_Cl_2_/EtOAc 10/1 (v/v). The collected product fractions were combined and concentrated to dryness under reduced pressure to furnish **2** as a yellow residue. Dipyrrin **2** was isolated after crystallization from CH_2_Cl_2_/*n*-C_6_H_14_ (1/4 cm^3^) at 4 °C as 1.5 mg of yellow crystals (yield 76 %). M.p.: 178 °C; UV/Vis (CH_2_Cl_2_): *λ*_max_ (log *ε*) = 327 (3.72), 436.5 (4.42) nm; fluorescence emission (CH_2_Cl_2_, *c* = 4 × 10^−6^ mol/dm^3^, excited at 436 nm, rel. intensity): 530 nm (1.00); fluorescence excitation (obs. for 530 nm, rel. intensity): 437 (1.00), 330 (0.40) nm; ^1^H NMR (300 MHz, CDCl_3_): *δ* = 1.35 (s, CH_3_ of *t*-Bu), 3.17 (s, H_2_C3^1^, H_2_C7^1^), 4.10 (s, H_2_C2^1^, H_2_C8^1^), 7.06 (d, *J* = 1.7 Hz, HC52, HC52′), 7.58 (s, HC1, HC9), 7.61 (t, *J* = 1.7 Hz, HC54), 12.76 (br s, HN11) ppm; FAB-MS: *m*/*z* (%) = 515.2 (14), 514.2 (33), 513.1 (100, [M+H]^+^, calcd. for C_27_H_32_N_2_O_4_S_2_ (512.18), 450.1 (10), 449.2 (27), 448.2 (32, [M–SO_2_]^+^), 386.2 (15), 385.2 (52), 384.2 (75, [M–2SO_2_]^+^).

### *Bis(5*-*(3,5*-*di*-*tert*-*butylphenyl)*-*di(β,β′*-*sulfoleno)pyrrinato) Zn*-*complex* (**3**, C_54_H_62_N_4_O_8_S_4_Zn)

Dipyrrin **2** (2 mg, 3.8 μmol) was dissolved in 2.7 cm^3^ CH_2_Cl_2_ and the solution of 15 mg Zn(OAc)_2_ 2H_2_O (68.4 μmol, 18 eq.) in 0.3 cm^3^ MeOH was added. After 5 min, the reaction mixture was diluted to 15 cm^3^ with CH_2_Cl_2_ and washed with saturated aq. NaHCO_3_ (3 × 10 cm^3^). The organic layer was filter through a plug of dry cotton wool and evaporated to dryness under reduced pressure to give a red residue. After crystallization in CH_2_Cl_2_/*n*-C_6_H_14_ (1.5/4 cm^3^) at 4 °C, 2 mg of **3** were obtained as pink-red crystals, yield is 94 %. M.p.: >195 °C (decomp.); UV/Vis (CH_2_Cl_2_): *λ*_max_ (log *ε*) = 242 (4.07), 357.5 (3.81), 385sh (3.66), 463sh (4.51), 487 (4.82) nm; fluorescence emission (CH_2_Cl_2_, *c* = 4 × 10^−6^ mol/dm^3^, excited at 487 nm, rel. intensity): 505 nm (1.00); fluorescence excitation (obs. for 505 nm, rel. intensity): 486 (1.00), 461sh (0.49), 355 (0.10) nm; ^1^H NMR (300 MHz, CDCl_3_): *δ* = 1.40 (s, CH_3_ of *t*-Bu), 3.11 (s, H_2_C3^1^, H_2_C7^1^), 4.09 (s, H_2_C2^1^, H_2_C8^1^), 7.11 (d, *J* = 1.7 Hz, HC52, HC52′), 7.54 (s, HC1, HC9), 7.66 (t, *J* = 1.7 Hz, HC54) ppm (due to the symmetric structure, only half molecular chemical shifts were labeled here); FAB-MS: *m*/*z* = 1087.2 ([M+H]^+^, calcd. for C_54_H_62_N_4_O_8_S_4_Zn 1086.27), 1023.3 ([M–SO_2_+H]^+^), 894.4 ([M–3SO_2_]^+^), 830.4 ([M–4SO_2_]^+^).

Crystallographic data: C_54_H_62_N_4_O_8_S_4_Zn × CH_2_Cl_2_ × 0.75 C_6_H_14_, formula weight: 1238.24; temperature 233(2) K; radiation wavelength 0.71073 Å; crystal system triclinic; space group *P*-1 (no. 2); unit cell dimensions *a* = 18.5998(4) Å, *b* = 18.9173(3) Å, *c* = 21.6741(5) Å, *α* = 98.771(1)°, *β* = 104.394(1)°, *γ* = 109.204(1)°; volume 6743.1(2) Å^3^; *Z* = 4; density (calculated) 1.220 g/cm^3^; absorption coefficient 0.618 mm^−1^; crystal size 0.45 × 0.25 × 0.03 mm^3^; *F*(000) 2606; theta range for data collection 1.335°–24.145°; index ranges −21 ≤ *h* ≤ 21, −21 ≤ *k* ≤ 21, −24 ≤ *l* ≤ 24; reflections collected 36397; independent reflections 21231 [*R*(int) = 0.0379]; completeness to theta (24.145°) 98.5 %; absorption correction none; refinement method Full-matrix least-squares on *F*^2^; data/restraints/parameters 21231/29/1489; goodness-of-fit on *F*^2^ 1.032; final *R* indices [*I* >2*σ*(*I*)] *R*_1_ = 0.0769, *wR*_2_ = 0.1998; *R* indices (all data) *R*_1_ = 0.1148, *wR*_2_ = 0.2209. Crystallographic data of **3** (Zn-(**2**)_2_) (excluding structure factors) have been deposited with the Cambridge Crystallographic Data Centre as supplementary publication number CCDC no. 1453954. Copies of the data can be obtained free of charge from the Cambridge Crystallographic Data Centre via http://summary.ccdc.cam.ac.uk/structure-summary-form.
